# Inflammatory Biomarkers, Microbiome, Depression, and Executive Dysfunction in Alcohol Users

**DOI:** 10.3390/ijerph17030689

**Published:** 2020-01-21

**Authors:** Mary Rodríguez-Rabassa, Pablo López, Raphael Sánchez, Cyanela Hernández, Cesarly Rodríguez, Ronald E. Rodríguez-Santiago, Juan C. Orengo, Vivian Green, Yasuhiro Yamamura, Vanessa Rivera-Amill

**Affiliations:** 1 Center for Research Resources, Ponce Health Sciences University-Ponce Research Institute, Ponce, PR 00716-2348, USA; marodriguez@psm.edu (M.R.-R.); plopez@psm.edu (P.L.); rsanchez@psm.edu (R.S.); erodriguez15@stu.psm.edu (R.E.R.-S.); bonyamam@gmail.com (Y.Y.); 2 Clinical Psychology Program, Ponce Health Sciences University-Ponce Research Institute, Ponce, PR 00716-2348, USA; chernandez@stu.psm.edu (C.H.); crodriguez@stu.psm.edu (C.R.); 3 Public Health Program, Ponce Health Sciences University-Ponce Research Institute, Ponce, PR 00716-2348, USA; jorengo@psm.edu (J.C.O.); vgreen@psm.edu (V.G.)

**Keywords:** executive dysfunction, depression, alcohol, cytokines, microbiome

## Abstract

Alcohol-related disorders (ARD) are highly prevalent among Latin American-Caribbean countries. Mental disorders are common comorbidities in individuals with ARD. However, the etiology of the association between ARD and mental disorders remains unclear. We examined the association of inflammatory cytokines, microbiome, and other biomakers with measures of depression, social anxiety, and executive functions. We observed a significant increase in cytokine and chemokine expression levels in saliva and plasma in the alcohol group (AG) samples. Also, the salivary bacterial composition in the AG revealed an abundance of *Prevotella*. Depression symptomatology was markedly higher in the AG, but social anxiety levels were negligible. AG also exhibited executive dysfunctions, which negatively correlated with increased plasma levels of pro-inflammatory cytokines and increased salivary concentrations of *Prevotella* bacteria. Our study suggests that chronic alcohol use correlates with executive dysfunction, immune system dysregulation, and dysbiosis of the salivary microbiota. Additional studies are needed to understand the role of the microbiome and inflammation in alcohol use and mental comorbidities.

## 1. Introduction

Alcohol drinking is an important social activity in Latin American-Caribbean (LAC) culture; as a result, alcohol-related disorders (ARD) are highly prevalent among LAC countries [1,2,3]. A 2008 survey conducted in the Puerto Rican population aged 15–74 years reflected that 76.8% had used alcohol at least once during the individual’s lifetime, and 48.8% reported using alcohol in previous 12 months [4]. According to the study, 12.1% of the participants reported suffering from alcohol abuse and 4.8% had alcohol dependence. A 2012 survey conducted with licensed drivers aged 16 years and older revealed that 22% of the participants admitted having driven a motor vehicle after consuming alcohol. Also, 33% of driving fatalities reported in Puerto Rico in 2016 were attributed to alcohol use; 66% of them also presented a speeding factor [5]. These findings suggest impairments in drivers’ executive functions (i.e., cognitive modulation of goal-directed behaviors) [6]. In addition to the aspects above, multiple problems arise because of excessive alcohol use such as progression to illegal drugs, consumption of multiple substances, job loss, domestic or interpersonal violence, cancer, cirrhosis, and mental disorders.

Mental disorders, such as anxiety and depression, are common comorbidities in individuals with ARD [7,8,9,10,11]. However, the etiological nature of the association is still disputed [12,13,14,15]. Indeed, the 2008 survey previously mentioned found that almost one-third (31.4%) of the participants with alcohol dependence had comorbid depression and anxiety disorders [4]. In recent decades, research made significant progress in understanding how emotional or physical stresses cause depression and anxiety. Leclercq et al. identified the alcohol-induced dysbiosis of gut microbiota and ensuing inflammatory responses as the likely causes of depressive disorders and alcohol craving [16]. A significant body of literature supports a direct association between dysbiosis of gut microbiota and inflammatory responses [17,18,19,20,21,22], and hence mental disorders [23,24,25,26,27,28,29,30]. Research made significant progress in understanding how emotional or physical stresses cause depression and anxiety. Furthermore, depression is associated with an increase of pro-inflammatory cytokines (e.g., interleukin-6) [31]. Alcohol use increases endotoxemia [32,33,34,35], while chronic alcohol use alters oral [36,37,38] as well as gut microbiota [38,39,40,41,42,43] and the executive functions of the brain [44,45,46,47].

The objective of this study is to determine the association between inflammation and microbiome composition in alcohol use. We also explored the possible associations among some of the essential blood and saliva components (cytokines, insulin, leptin, cortisol, and vitamin D) with measures of depression, social anxiety, and executive functions.

## 2. Materials and Methods

This case-control pilot study was conducted in accordance with the Declaration of Helsinki, and the protocol was approved by the Institutional Review Board of the Ponce Medical School Foundation, Inc. (IRB approval no: 140718-MR). All participants signed an informed consent before sample collection and completion of study questionnaires. The study involved the collection of measures of participant’s alcohol consumption, executive functions, depression symptomatology, social anxiety, inflammatory biomarkers, vitamin D, leptin, insulin, cortisol, and salivary microbiome as described below.

### 2.1. Study Participants

Fifty participants were recruited in response to the study flyer placed in the medical and psychiatric clinic of the Ponce Health Sciences University (PHSU), located in the southern part of Puerto Rico. Participants were included in the study if they were ≥21 years old; possessed the mental capacity to understand the purpose, risk, and benefits of participating in the study; and were able to answer the study questionnaires and consent to the collection of saliva and blood. The exclusion criteria were having, at the time of recruitment, obvious oral or skin infections, which required treatment; being judged to be under the influence of substances; and requiring immediate medical attention for any reasons. Participants were classified into two groups, alcohol or control, based on the individual’s reported alcohol consumption, using the Alcohol Use Disorders Identification Test (AUDIT) scale [48]. The alcohol group (AG) included 30 participants (18 males and 12 females) and the control group (CG) included 20 participants (10 males and 10 females).

Each participant was offered a brief explanation of the purpose of the study, risks and benefits involved, their right to not participate in the study, the biological samples to be collected, and tests to be performed. After informed consent was discussed and voluntarily signed, the study staff assessed the participant’s alcohol consumption during the past 12 months using the AUDIT scale [48]. Cognitive ability was measured using the NIH Toolbox Cognitive Battery Test [49]. Depression symptoms were assessed using the Patient Health Questionnaire-9 (PHQ-9) [50], and social anxiety was measured using the Liebowitz Social Anxiety Scale [51]. Blood and saliva samples were collected at the time of evaluation, with most samples collected in the morning.

The participants provided information about their age, level of education, occupation, civil status, mental and physical conditions, and psychiatric history. They also answered questions regarding prescribed drugs, alcohol, smoking and drugs habits, sleep difficulties, and relationships with others. The information was carefully collected to prevent association with the participant’s identity. All study analyses were performed using coded de-identified participant data.

### 2.2. Assessment of Alcohol Consumption

Participants were asked to answer the questionnaire AUDIT [48] to evaluate their alcohol consumption. AUDIT is a 10-item questionnaire that explores the amount and frequency of drinking, alcohol dependence, and personal and social consequences caused by alcohol consumption during the past 12 months; each item has five answer options coded 0 through 4 [48]. A cutoff score of 8 points was chosen for defining participants’ classification into either the control or alcohol group. Scores of 8–15, 16–19, ≥20 are considered as alcohol use in excess, harmful and hazardous drinking, and possible alcohol dependence, respectively. Participants were also asked to provide a breath sample to estimate the breath alcohol concentration. A calibrated BACtrack Pro S80 Breathalyzer [52] was used for this purpose.

### 2.3. Assessment of Executive Functions

The NIH Toolbox Cognition Battery [53] is a computer-based adaptive test that assesses cognition in the following areas: executive function, attention, episodic memory, language, processing speed, and working memory. The test generates several scores, including fully adapted national standard scores, which are used to classify participants’ cognitive abilities based on the expected mean (100 points) and standard deviation (15 points). This study focused on the executive functions measured using the Flanker Inhibitory Control and Attention Test and the Dimensional Change Card Sort Test. These tests are related to the ability to inhibit automatic response tendencies that may interfere with achieving a goal and the ability to shift among multiple aspects of a strategy or task, respectively [53].

### 2.4. Assessment of Social Anxiety

The Liebowitz Social Anxiety Scale Self-Report (LSAS-SR) is a valid and reliable scale [51] for determining if an individual feels fear or anxiety about his/her diverse social situations and if he/she tries to avoid those situations. It has 24 items, each one of which is rated separately for fear/anxiety; (0 = none, 1 = mild, 2 = moderate, 3 = severe) and avoidance behavior (0 = never, 1 = occasionally, 2 = often, 3 = usually). Scores of <55, 55–65, 65–80, 80–95, >95 are considered as no to negligible, moderate, marked, severe, and very severe levels of social anxiety, respectively.

### 2.5. Depression Symptomatology

Depression symptoms were assessed using the nine-item Patient Health Questionnaire (PHQ-9). This self-report scale is a valid and reliable measure of depression severity [50]. Each response is scaled by how often the described symptom bothered the individual during the last two weeks (0 = not at all, 1 = several days, 2 = more than half the time, 3 = nearly every day). Total scores were used to assess depression severity as follow; ≤4 (minimal), 5–9 (mild), 10–14 (moderate), 15–19 (moderately severe), and ≥20 (severe, major depression).

### 2.6. Collection of Biological Samples

Approximately 5 mL of saliva were collected from each participant. Initially, they were asked to rinse their mouth twice with room-temperature water. After five minutes, they were asked to spit into a conical 50 mL-volume, sterile conical centrifuge tube. Saliva samples were stored in aliquots at −85 °C until analysis [54].

A licensed medical technologist, experienced in phlebotomy, aseptically collected about 10 mL of venous blood into three separate EDTA-containing blood collection tubes (BD Biosciences, Franklin Lakes, NJ, USA). The blood samples were centrifuged for plasma separation and stored in aliquots at −85 °C until analysis.

### 2.7. Cytokines, Chemokines, Leptin, Insulin, and Cortisol Assays

We assessed the concentration of pro-inflammatory and anti-inflammatory cytokines and chemokines in saliva and plasma samples using the MILLIPLEX^®^ MAP Human Cytokine/Chemokine Magnetic Bead Panel Premixed 39-plex Immunology Multiplex Assay kit (EMD Millipore, Chicago, IL, USA) following manufacturer’s instructions as previously described [55]. Briefly, a premixed cytokine bead suspension was incubated with undiluted saliva or plasma. A minimum of 50 microsphere events were acquired for each cytokine using the Luminex MAGPIX^®^ System with the xPONENT^®^ software (Luminex Corp., Austin, TX, USA). The median fluorescent intensity (MFI) was obtained for each cytokine using a five-parameter curve-fitting method of the program, and the software automatically calculated each cytokine concentration. Plasma and salivary concentrations of leptin, insulin, and cortisol were assayed using the Millipore Human Magnetic Bead Panel kit, following the manufacturer’s instructions.

### 2.8. Microbiome Analysis by Next Generation Sequencing

We performed the metagenomic analysis of the bacterial gene coding for the16S ribosomal RNA according to the Illumina MiSeq 16S metagenomics sequencing library preparation procedure (part #15044223 rev. A; Illumina Inc., San Diego, CA, USA). Bacterial DNA was purified from 150 L of saliva using the QIAGEN blood DNA extraction kit following the manufacturer’s recommendations (QIAGEN, Venlo, Limburg, The Netherlands). Once the DNA was purified, the conserved 16S V3 and V4 region was PCR amplified using the FastStart PCR master mix (Roche Diagnostics, Indianapolis, IN). The PCR conditions were as follows: 95 °C for 3 min, followed by 25 cycles (95 °C for 30 s, 55 °C for 30 s, 72 °C for 30 s), and a final elongation step at 72 °C for 5 min. The following primer pair suggested by Klindworth et al. [56] was used for the PCR: FWD: 5′TCGTCGGCAGCGTCAGATGTGTATAAGAGACAGCCTACGGGNGGCWGCAG3′ and REV: 5’GTCTCGGTGGGCTCGGAGATGTGTATAAGAGACAGGACTACHVHHHTATCTAATCC3′.

Illumina AMPure XP beads were used to purify the amplicons. The concentration of the amplicons was adjusted to a concentration of 0.2 ng DNA/µL. We performed the Index PCR using the Illumina Nextera DNA preparation kit (catalog number FC-121-1031) under the same conditions as described above, except that the Nextera index 1 and index 2 primers were used in place of the forward and reverse primer pair described above. AMPure XP beads were used to purify the Nextera amplicons. The library data was then acquired using the Illumina MiSeq Reporter software. The acquired data were extracted, decompressed, and then analyzed using the QIIME software in a Linux platform (Ubuntu), as previously described by Caporaso et al. [57]. Sequences were de-multiplexed, quality filtered, and a Seqs.fna file was generated. This file was then used to create an operational taxonomic unit (OTU) table. The OTU table provided the taxonomic composition of the microbe community. Statistical analyses were then performed using the OTU table data.

The sequence data were submitted to NCBI BioProject (http://www.ncbi.nlm.nih.gov/bioproject) under accession number PRJNA520863.

### 2.9. Vitamin D Assay

Plasma and salivary concentration of vitamin D were assayed as its hydroxyl form using ENZO 25(OH) Vitamin D^®^ ELISA kit (ENZO, Farmingdale, NY, USA), following the manufacturer’s instructions. Determination of optical density was performed in a BioTek Synergy HT (BioTek, Winooski, VT, USA) set to 450 nm.

### 2.10. Dietary Assessment

Nutrient data were collected using the Food Frequency Questionnaire (FFQ), developed by the Nutrition Assessment Shared Resource (NASR) of the Fred Hutchinson Cancer Research Center (FHCRC). Participants reported the frequency of consumption and portion size of 125 foods and beverages using a Likert-type scale referencing food intake patterns during the last month. The FFQs were processed for nutrient analysis through the University of Minnesota Nutrition Data Systems for Research (NDSR) software [58,59,60].

### 2.11. Data Analyses

Statistical analyses were performed using IBM SPSS (v 24.0, Armonk, NY, USA). Descriptive statistics included means, medians, standard deviation, and interquartile ranks; these were assessed for all continuous variables. Bivariate analysis was used to analyze differences in sex, age, and education. Chi-square was used to compare differences between groups; *p*-values < 0.05 were considered statistically significant. Mann–Whitney U tests were performed to compare the differences between two independent groups. Multiple logistic regression adjusted odds ratio was used to assess the association between alcohol use and inflammation, depression, anxiety, and executive functions. Spearman Rho was used to assess correlations among depression, social anxiety, executive functions, microbiota, and inflammatory markers taking into consideration the source of the sample (saliva or plasma).

The microbiota structure and statistical comparison of the metagenomic samples were evaluated using the MicrobiomeAnalyst online platform [61]. Briefly, data were filtered to improve downstream statistical analyses by using a minimum count at four with prevalence and low variance (based on inter-quantile range) at 20% and at 10%, respectively. Normalization was performed by rarefying to the minimum library size (2846) and scaling to the cumulative sum scaling (CSS) method. The abundance profiling (phylum/genus) were based on the sum of their counts across the groups. The core microbiome analysis, which is the evaluation of a group of taxa that were in a high fraction of the population, was performed at a sample prevalence of 20% and a relative abundance of 0.2%. The beta-diversity analysis was performed by using the Bray-Curtis index distance method with a permutational MANOVA (PERMANOVA) statistical method.

The alpha-diversity profiling and significance testing were evaluated by using Shannon metrics and Mann–Whitney/Kruskal–Wallis, respectively. The univariate statistical comparison at the genus level was evaluated by using the Mann–Whitney U Test statistical method with an adjusted *p*-value cutoff of 0.05. The linear discriminant analysis effect size (LEfSe) and random forest methods were used to perform biomarker analysis [62,63]. The LEfSe, which employs a non-parametric Kruskal–Wallis rank-sum test, was obtained with an adjusted *p*-value cutoff of 0.05 and a log of the linear discriminant analysis (LDA) score of 1. Random forest analysis was at the genus level with 500 bootstraps, randomness setting on, and seven numbers of predictors to try.

## 3. Results

### 3.1. General Characteristics of the Study Participants

Table 1 summarizes the general characteristics of the study participants in the alcohol (AG) and control (CG) groups. The self-descriptive information was obtained through individual interviews. The average age of the participants was 41.6 years old for the alcohol group and 37.9 years old for the control group. All participants were of Hispanic ethnicity. The majority of study participants were unemployed (36.7% of AG, 50.0% of CG) or retired (13.3% of AG and 20.0% of CG). The AG (43.3%) had more participants with lower education than the CG (10.0%). There was a higher number of participants in the AG who reported smoking as compared to the CG (63.3% vs. 10.0% in CG). Those who reported smoking also had higher risks of drinking: OR = 15.5 (95% CI: 3.01–80.04; *p* < 0.01). Both groups reported having medical conditions (60.0% for AG; 55.0% for CG) but not statistically significant differences were found. The most prevalent medical conditions were hypertension (23.3% for AG; 45.0% for CG) and hypothyroidism (6.6% for AG; 20.0% for CG). The participants in both groups were receiving medication to treat these conditions. Another medical condition reported was asthma (13.3% for AG; 5.0% for CG) although symptoms were absent at the time of assessment.

We also explored the history of psychiatric treatment. The AG showed a higher prevalence of depression (30% vs. 10%) and anxiety (37% vs. 10%) disorders than the CG. Participants from both groups reported using antidepressants or anxiolytics. Most of the study participants from the AG reported having a sleep disorder (80% vs. 35% from the CG); alcohol consumption increased the odds of also having a sleep disorder OR = 7.4 (95% CI: 2.1–26.7, *p* < 0.05). Only 7% of the AG reported a diagnosis of alcohol dependence disorder. However, most of AG participants (73%) reported having a family history of alcohol dependence, which increases the odds of drinking alcohol OR = 3.3 (95% CI: 1.01–11.11, *p* < 0.05). The mean of breath alcohol concentration for the AG was 0.01 (SD = 0.03), whereas the mean for the CG was 0. AUDIT median score for the AG was 18 (IQR = 15.5–28.0). Most of the participants in AG showed a pattern of harmful and hazardous drinking (33%) or possible alcohol dependence status (43%), according to AUDIT scores.

### 3.2. Depression and Social Anxiety Levels of the Study Participants

Table 2 summarizes the scores and severity levels of depression and anxiety, as measured by the study instruments. The PHQ-9 median scores reflected statistically significant differences among groups; the AG showed more depression symptoms than the CG (8.5 vs. 4; *p* ≤ 0.01). A considerable portion of the participants from both groups reported having minimal or mild depression (57% and 85% for AG and CG, respectively); 43% of the AG versus 15% of CG indicated symptoms corresponding to moderate to severe depression. However, LSAS-SR scores showed that the majority of study participants have a negligible level of social anxiety.

### 3.3. Cognitive and Executive Function Abilities

Participants’ cognitive and executive function abilities were measured using the NIH Toolbox Cognition Battery test. As shown in Table 3, participants from the AG exhibited significantly lower median standard scores in the NIH Toolbox Cognition Composite Score (75.5 vs. 100.0; *p* ≤ 0.001). Statistically significant differences among groups were also detected in measures of executive functions. The AG showed lower median standard scores in Flanker inhibitory (82.5 vs. 103.0, *p* ≤ 0.001) as well as in dimensional change card sort (95.0 vs. 107.0; *p* ≤ 0.001). These scores were re-coded in two intervals as follows: <85 and ≥85. Our results show that drinking alcohol increases the odds of having lower cognition ability (OR = 21.2; 95% CI: 3.9–113.9; *p* ≤ 0.001). Also, drinking alcohol increases the odds of lower abilities in executive functions: Flanker inhibitory score OR = 8.2 (95% CI: 1.9–35.1; *p* ≤ 0.01), and dimensional change card sort OR = 1.9 (95% CI: 1.4–2.5; *p* ≤ 0.05).

### 3.4. Plasma and Salivary Levels of Cytokines

We determined the concentrations of the following cytokines in plasma and saliva: TNF-α, IL-12p70, MDC, IL-10, IFN-γ, TNF-β, IL-1β, IL-5, IL-2, IL-6, IL-17A, IL-4, IL-1RA, IL-13, IL-7, and GM-CSF among other analytes. The median concentration levels of plasma and saliva levels of cytokines with statistically significant differences are presented in Table 4**.** The AG showed significantly lower concentrations of salivary IL-12p70, and IL5, and significantly higher concentrations of TNF-β, IL-2, IL-4, and IL-13. Also, plasma levels of MDC were significantly higher in the AG.

### 3.5. Plasma and Salivary Levels of Insulin, Leptin, Cortisol, and Vitamin D

Analysis of plasma and saliva levels of insulin, leptin, cortisol, and vitamin D revealed no statistical difference of leptin, insulin and cortisol between the AG and CG groups (Table 5). Interestingly, the CG exhibited lower levels of vitamin D in plasma (45.8 pg/mL and 18.4 pg/mL for AG and CG, respectively). Vitamin D is significantly reduced in saliva for the AG (*p* = 0.001). In contrast, the blood level of vitamin D in the AG is significantly higher than the CG (*p* = 0.01).

**Figure 1:**
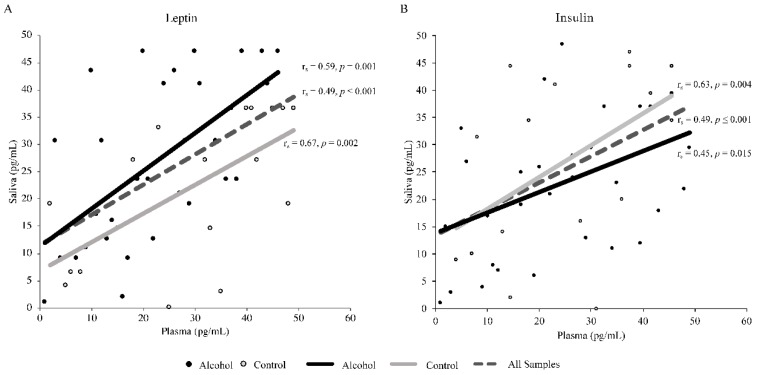
Spearman’s rank correlation between plasma and salivary leptin levels (**A**) and plasma and salivary insulin levels (**B**). The analyses included a total of 49 samples, of which 29 were from the alcohol group and 20 from the control group.

A significant positive correlation was detected between plasma and salivary leptin levels in the total sample as well as in AG and CG (r_s_ = 0.57, *p* ≤ 0.001; r_s_ = 0.59, *p* = 0.001; and r_s_ = 0.67, *p* = 0.002, respectively; Figure 1A). We also found a significant positive correlation between plasma and salivary insulin levels (r_s_ = 0.49, *p* ≤ 0.001; r_s_ = 0.45, *p* = 0.015; and r_s_ = 0.63, *p* = 0.004, respectively; Figure 1B).

### 3.6. Salivary Microbiome Assessment

**Figure 2:**
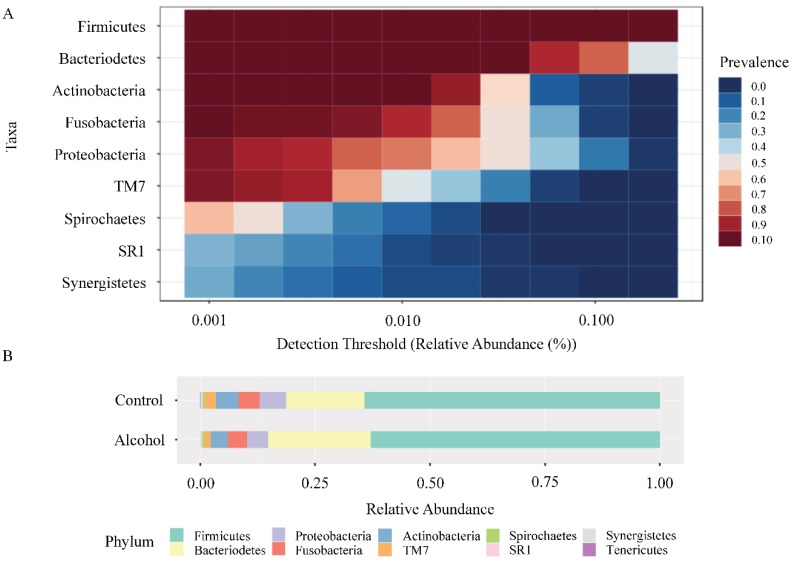
Microbial communities identified at the phylum level. (**A**) Heatmap of the core microbiome analysis to identify core taxa at phylum level. A total of 49 samples are included in the visual representation of the taxonomic relative abundance at phylum level. The *y*-axis represents the prevalence level of core features across the detection threshold (relative abundance) range on *x*-axis. The variation of prevalence of each phylum is indicated by a gradient of color from blue (decreased) to red (increased). (**B**) Taxonomic composition of alcohol group and control group at the phylum level. Visual exploration to evaluate taxonomic composition of community through direct quantitative comparison of relative abundances was performed by using stacked bar chart.

A total of 1,285,157 sequences were obtained after sequence filtering, with an average of 26,227 (Max.: 206,893/Min.: 2846). According to the data obtained, there were ten microbial communities identified at the phylum-level: *Firmicutes* (62.5% AG, 64.5% CG), *Bacteroidetes* (22.6% AG, 17.2% CG), *Proteobacteria* (4.5% AG, 5.5% CG), *Fusobacteria* (4.2% AG, 4.6% CG), *Actinobacteria* (3.8% AG, 4.8% CG), *TM7* (1.4% AG, 2.3% CG), *Spirochaetes* (0.5% AG, 0.5% CG), *SR1* (0.3% AG, 0.2% CG), *Synergistetes* (0.2% AG, 0.4% CG), and *Tenericutes* (0.05% AG, 0.1% CG) (Figure 2A). Nevertheless, no significant differences were observed between AG and CG at this level (Figure 2B).

**Figure 3:**
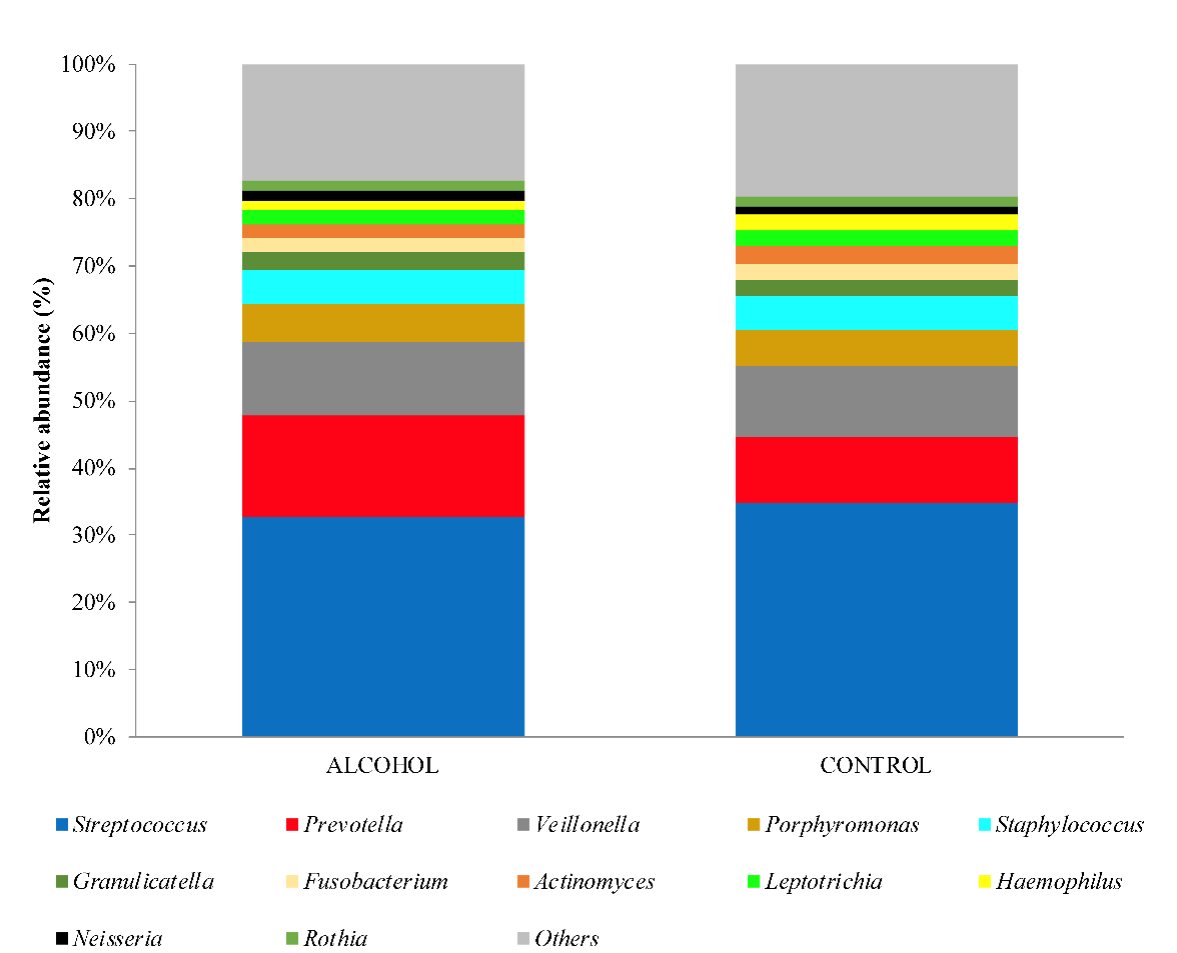
Stacked bar chart showing the taxonomic composition and relative abundance of the 13 major genera observed between the alcohol group and the control group. Taxa with very low read counts was collapsed into others category.

Meanwhile, at genus level, *Streptococcus* was the predominant taxon in both groups (33% AG, 35% CG) followed by *Prevotella* (15% AG, 10% CG), *Veillonella* (11% AG, 10% CG), *Porphyromonas* (6% AG, 5% CG), *Staphylococcus* (5% AG, 5% CG), *Granulicatella* (3% AG, 2% CG), *Fusobacterium* (2% AG, 2% CG), *Actinomyces* (2% AG, 3% CG), *Leptotrichia* (2% AG, 2% CG), *Haemophilus* (1% AG, 3% CG), *Neisseria* (1% AG, 1% CG), *Rothia* (1% AG, 2% CG), and others (Figure 3).

**Figure 4:**
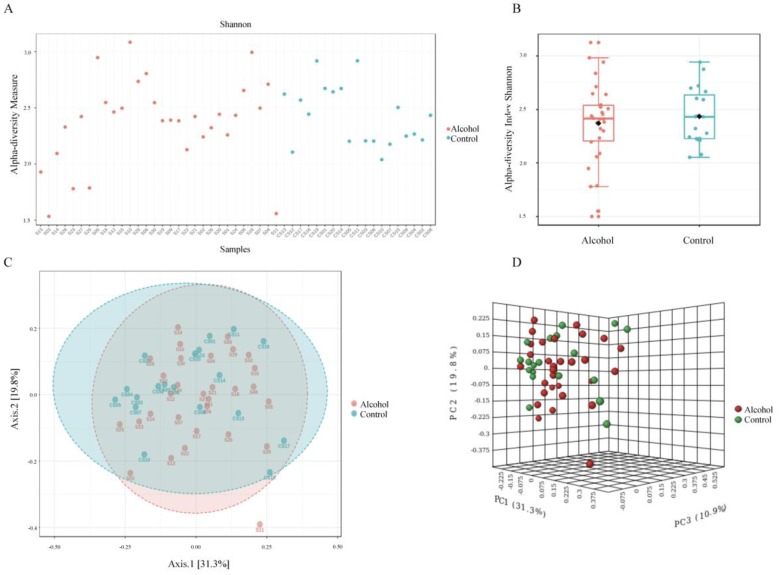
Alpha and beta diversity profiling and significance testing. The Shannon alpha-diversity analysis at genus level was performed in filtered data input by using Mann–Whitney/Kruskal–Wallis statistical method. The samples (**A**) or the groups (**B**) were represented on *x*-axis and their estimated diversity on *y*-axis. Each sample or boxplot are colored based on alcohol consumption status (alcohol users = red, control = blue). The beta-diversity analysis at genus level by using the non-phylogenetic Bray–Curtis index distance method was using to stablish the changes in abundance of taxa present in the dataset. Principle coordinate analysis (PCoA) was used to visualize these matrixes in 2 (**C**) or 3-D plot (**D**). Each point in the graph (alcohol users = red, control = green) represents the entire microbiome analyses of a single sample. The statistical significance of the clustering pattern was evaluated by using Permutational MANOVA (PERMANOVA).

**Figure 5:**
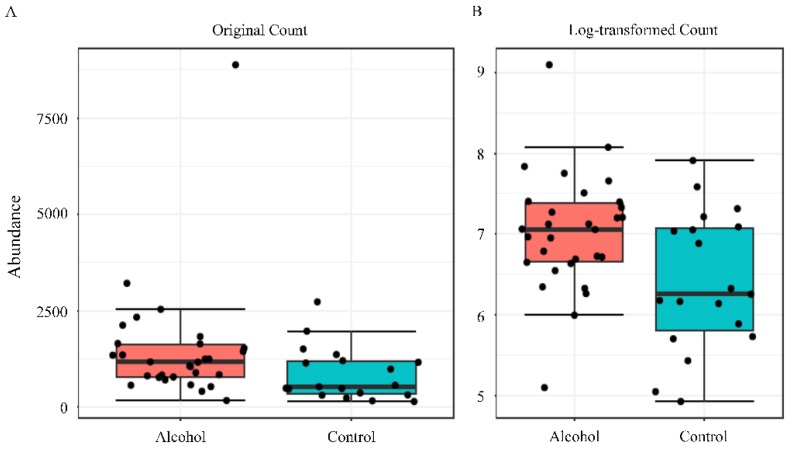
Differential abundance analysis was performed to evaluate *Prevotella* genus by using univariate statistical composition. Each boxplot, which visualization are in original count (panel **A**) or log-transformed count (panel **B**), represents the abundance distribution of *Prevotella* genus. The Mann–Whitney/Kruskal–Wallis method with adjusted *p*-value cut-off of 0.05 was used to perform statistical analyses.

**Figure 6:**
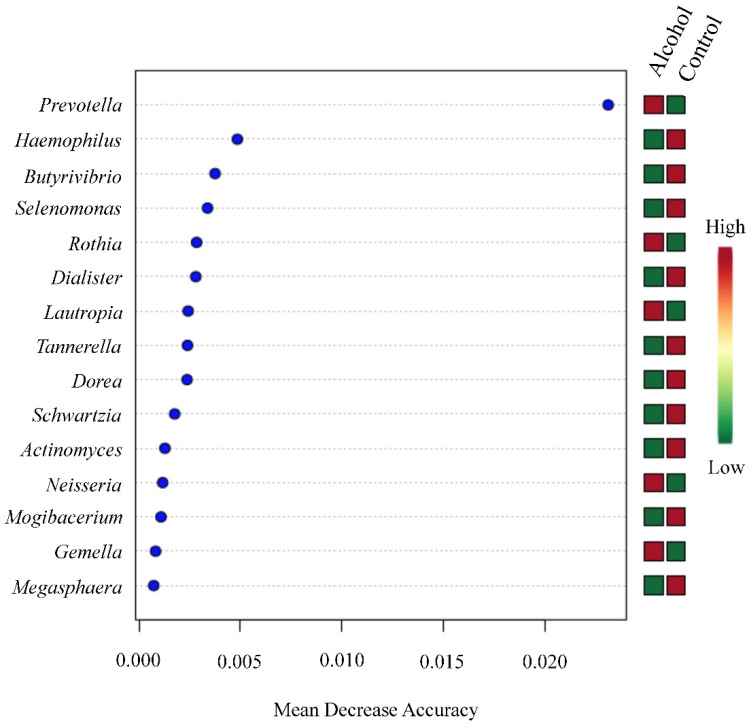
Random forests ranked by contributions to classification accuracy (mean decreased accuracy). The random forest analysis, which is an algorithm suitable method for high dimensional data analysis, was performed by using the ensemble of classification trees by random feature selection from a bootstrap sample at each branch. The 15 more important genera were classified based in their mean decreased in accuracy (high = red, low = green).

**Figure 7:**
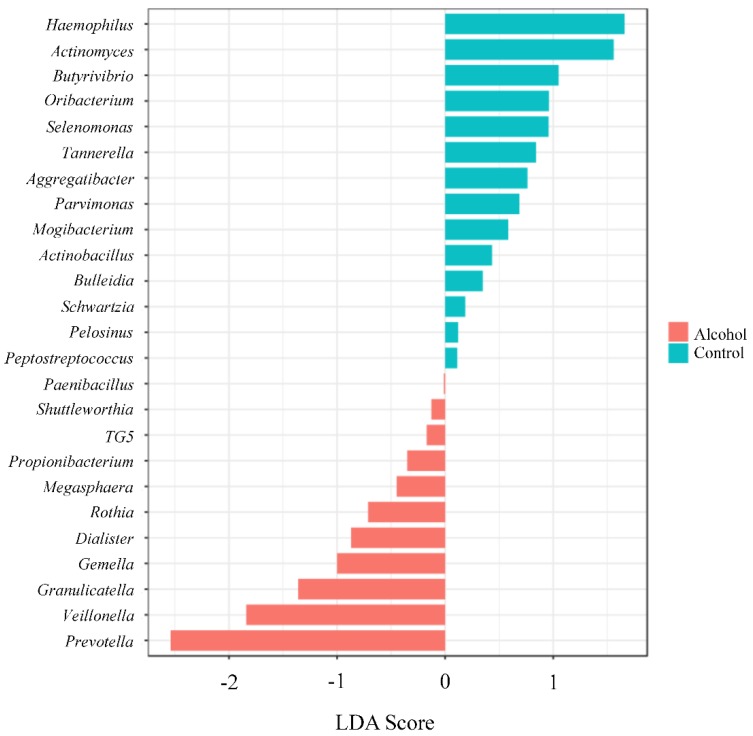
The differences between the groups were evaluated by using the linear discrimination analysis effect size (LEfSe). The LEfSe method, which is a biomarker discovery, uses Kruskal–Wallis sum test and linear discrimination analysis to identify significant difference abundance and estimated the effect size, respectively. The bar graph shows the LDA scores of (at most) top 25 significant bacteria. The colors (alcohol users = red, control = blue) represent which group was more abundant compared to the other group at genus level. The *p* value cut-off was 0.1 with an adjusted false discovery rate.

The Shannon alpha-diversity did not reach significant differences (*p* value= 0.94) (Figure 4, panels A and B) between the two populations evaluated. Similar finding was observed in beta-diversity analysis (*f* value = 0.9019, R-squared = 0.014) (Figure 4, panel C and D). Nevertheless, a significant difference between the groups was only observed in *Prevotella* genus by using Mann–Whitney U Test statistical method (*p* value = 0.015) (Figure 5). The Random forest prediction (Figure 6) and LEfSe evaluation (Figure 7) suggest that *Prevotella* genus might be a possible biomarker of alcohol use in saliva. 

### 3.7. Diet

Analysis of nutrients that participants consumed in the last month showed statistically significant differences in reports of alcohol intake (16.75 *g* for AG; 0.04 *g* for CG). Reports of other nutrients did not show statistically significant differences among groups (Supplementary Table S1).

### 3.8. Cytokines and Microbiome Correlations with Executive Functions

**Figure 8:**
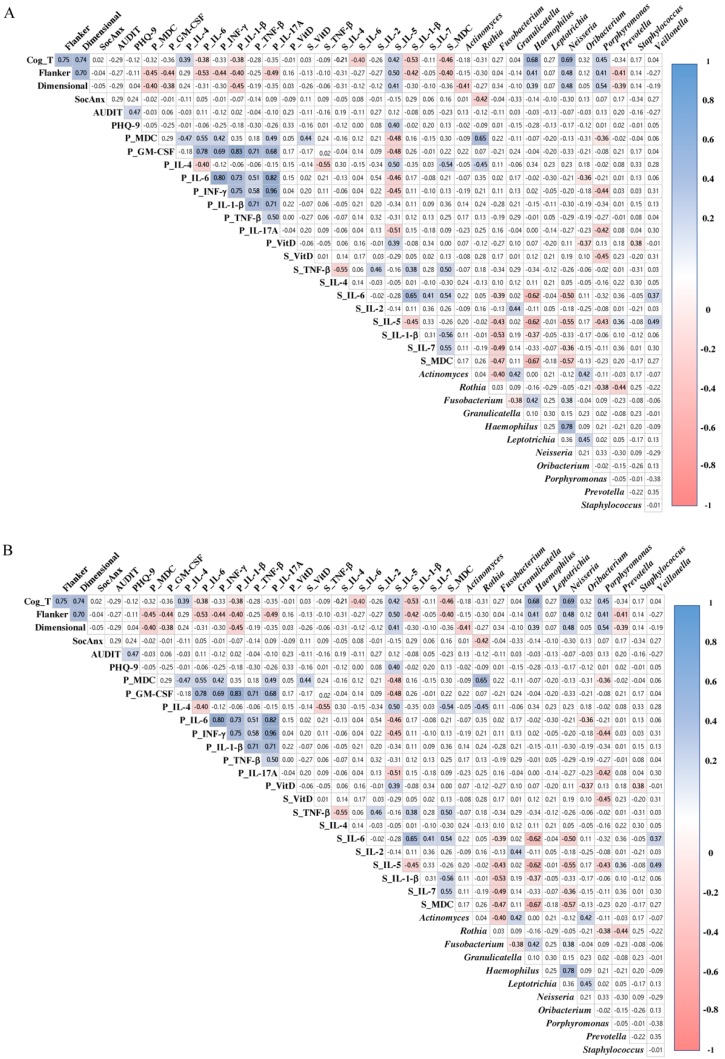
Spearman Rho correlation among depression, social anxiety, executive functions, microbiota, and inflammatory markers in alcohol group (**A**), and control group (**B**). White squares indicate no significant association; blue squares indicate significant positive association; red squares indicate significant negative associations. Color intensity reflects stronger associations as determine by the correlation coefficient value (*y*-axis); *p* < 0.05. Cog_T: cognitive composite score of the NIH toolbox cognition battery; Flanker: Flanker inhibitory control and attention test; Dimensional: dimensional change card sort test; SocAnx: scores in the Leibowitz Social Anxiety Scale; AUDIT_T: total score in the Alcohol Use Disorders Identification Test; PHQ9: scores in the nine-item Patient Health Questionnaire; P_MDC: levels of macrophage-derived chemokine in plasma; P_GM-CSF: levels of granulocyte-macrophage colony-stimulating factor in plasma; P_VITD: levels of vitamin D in plasma; S_VITD: levels of vitamin D in saliva; S_MDC: levels of macrophage-derived chemokine in saliva. Other cytokines/chemokines expressed in plasma and saliva are: P_IL-4, P_IL-6, P_INF-γ, P_IL-1β, P_TNF-β, P_IL-17A, S_TNF-β, S_IL-6, S_IL-2, S_IL-5, S_IL-1β, and S_IL-7. The following are bacteria found in saliva: *Actinomyces*, *Rothia*, *Fusobacterium*, *Granulicatella*, *Haemophilus*, *Leptotrichia*, *Neisseria*, *Oribacterium*, *Porphyromonas*, *Prevotella*, *Staphylococcus*, and *Veillonella*.

We found that salivary concentrations of *Prevotella* showed a significant negative correlation with Flanker (r_s_ = −0.408, *p* = 0.031) and Dimensional (r_s_ = −0.389, *p* = 0.037) in the AG but not in the CG (r_s_ = −0.001, *p* = 0.997; r_s_ = −0.074, *p* = 0.769, respectively; Figure 8). Higher expression of plasma concentrations of MDC and GM-CSF inversely correlated with both measures of executive functions in the AG: Flanker (r_s_ = −0.448, *p* = 0.017; and r_s_ = −0.444, *p* = 0.018, respectively), and dimensional (r_s_ = −0.402, *p* = 0.034; and r_s_= −0.383, *p* = 0.040, respectively). Other negative correlations were observed with Flanker scores in the AG with the following biomarkers: increased plasma IL-6 (r_s_ = −0.527, *p* = 0.004), plasma IFN-γ (r_s_ = −0.435, *p* = 0.021), plasma IL-17A (r_s_ = −0.49, *p* = 0.01), and salivary IL-1β (r_s_ = −0.402, *p* = 0.034). On the contrary, positive correlations were found in the following measures with Flanker in the CG: higher plasma TNF-β (r_s_ = 0.502, *p* = 0.029) and lower plasma GM-CSF (r_s_ = 0.474, *p* = 0.040). Lower expression of salivary IL-5 was positive correlated with Flanker (r_s_ = 0.500, *p* = 0.007) and dimensional (r_s_ = 0.413, *p* = 0.026) in the AG. Dimensional was positively correlated reduced salivary IL-7 in the CG (r_s_ = 0.497, *p* = 0.036).

Cognitive composite (CC) scores in AG were negatively correlated with salivary MDC and plasma and salivary IL-1β (r_s_ = −0.46, *p* = 0.01; r_s_ = −0.38, *p* = 0.05; and r_s_ = −0.53, *p* = 0.01, respectively). Higher expression of salivary and plasma IL-6 are inversely correlated with CC in the AG (r_s_ = −0.40, *p* = 0.04; r_s_ = −0.38, *p* = 0.05, respectively). On the contrary, the AG showed positive correlations of CC scores and plasma IL-4 (r_s_ = 0.39, *p* = 0.04) and salivary IL-5 (r_s_ = 0.42, *p* = 0.03). Lower expression of salivary TNF-β was negative correlated with CC scores in the CG (r_s_ = −0.54, *p* = 0.02).

## 4. Discussion

One of the main aims of this study was to examine plasma and saliva samples for inflammation and microbiome analyses in individuals with alcohol use living in Puerto Rico. The observed significant positive correlations between plasma and salivary IL-10, and between plasma and salivary IL-13 in alcohol users, suggest the possibility of using saliva as a non-invasive method for assessing these cytokines in this population. A recent study found significant correlations between the levels of IL-13 and IL-10, suggesting an interdependency between these two cytokines [64]. Periodontitis is a gum disease characterized by tissue breakdown caused by pathogenic bacteria of dental plaque, which activate the host immune-inflammatory response against these periodontal pathogens [65]. The AG showed higher salivary IL-10 and IL-13 than the CG. IL-10 has been identified to be increased in the process of a hangover after alcohol consumption [66]. IL-10 has also been associated with the development of periodontitis [67]. In periodontal diseases, IL-10 and IL-13 are involved in modulating the immune response and in the suppression of pro-inflammatory cytokines by monocytes/macrophages, respectively [68,69]. To our knowledge, there is little information on any associations between IL-13 in the oral cavity and alcohol use. Regardless, there have been studies that have found that alcohol consumption increases IL-13 signaling in airways [70,71].

On the other hand, these two anti-inflammatory cytokines, IL-10 and IL-13, have been identified as elevated in oral squamous cell carcinoma [72,73]. These cytokines have also been suggested as biomarkers for squamous cell carcinoma disease progression [72]. The results described above are not unexpected since alcohol consumption has been associated with oral squamous cell cancer [74]. Lastly, anti-inflammatory cytokines like IL-10 seem to be implicated in binge drinking. During binge drinking, IL-10 levels decreased in the basolateral amygdala. Interestingly, infusion of IL-10 decreased binge drinking behavior [75]. Further study of IL-13 could lead to defining what the role of this cytokine in the setting of alcohol use is.

Other significant positive correlations were observed between plasma and salivary levels of leptin and insulin in all samples and between groups. A recent review reported a significant correlation between serum and salivary insulin [76]. Similar correlations have been found between plasma and salivary leptin [77]; however, the need for additional studies to validate the use of saliva for these purposes is highlighted.

We also explored the associations among blood and saliva biomarkers (pro-inflammatory cytokines, vitamin D, insulin, leptin, and cortisol) with measures of executive functions depression, and social anxiety. Increased plasma levels of pro-inflammatory MDC, IL-6, IL-17A, INF-γ, and GM-CSF were negatively correlated to measures of executive functions in alcohol users, suggesting a possible role of these cytokines in their reduced ability to inhibit (restrain interferences for achieving a goal) and shifting (change attention between tasks). Besides, the negative correlation observed in salivary levels of IL-1β and measures of executive functions implies a protective factor: lower levels of these salivary biomarkers correlated with increased ability to inhibit and shifting.

Our results revealed that the AG exhibited executive dysfunctions, mainly a reduced ability to self-control and to shift responses based on rules or contingencies [78]. This finding supports previous studies reporting that alcohol use is linked to impairment in cognitive functioning [79,80]. However, it is crucial to take into consideration that deficits in executive functions may precede alcohol use [81] while conflictive results have been reported [82]. Consistent with the literature, co-morbid depression and anxiety [4,7,8,9,10,11] and polysubstance use [83] were more frequently reported in the AG. Both depression and polysubstance abuse are also linked to impairment in executive functions [79,84,85]. However, no social anxiety was present, which can be associated with the Latin American-Caribbean culture that promotes alcohol drinking as a social activity [86].

Chronic alcohol use has been reported to impair the immune response functions through effects on multiple organs such as the liver, lung, stomach, intestine, and brain [87,88]. According to our results, alcohol users showed increased plasma macrophage-derived chemokine (MDC). MDC, also referred to as chemokine CCL22, has a dual function: inflammatory and homeostatic; it stimulates Th2 response, Th2 cell migration, and T reg migration [89]. The Th2 cells produce IL-4, IL-5, IL-6, IL-9, IL-10, and IL-13 cytokines, and evoke strong antibody responses and eosinophil accumulation, but inhibit several functions of phagocytic cells [90]. Also, MDC has been associated with alcoholism, phobia, and interpersonal sensitivity [91].

The CG showed significantly increases levels of salivary IL-5 and IL-12p70 compared to the AG. It should be noted that although not statistically different, many of the CG participants reported having medical conditions that might impact their immunology profile. IL-5 plays a critical role in adaptive immune responses since it regulates eosinophils, cells that play a central role in the pathogenesis of allergic diseases, and induces cell maturation, survival, and activation [92,93]. IL-12p70 has a critical pro-inflammatory role in innate immunity and plays a vital role in the initiation of the protective immune response [92]. Interestingly, a recent study found increased levels of both cytokines after exposure to psychological stress in adults [94].

The AG presented significantly increased levels of salivary cytokines TNF-β, IL-2, IL-4, and IL-13, suggesting immune dysregulation in the oral cavity. Previous studies showed associations of increased risk for oral cancer with TNF-β polymorphisms genes and increased expression of salivary IL-4 and IL-13 [72,95]. Other studies comparing salivary cytokines of clinical samples and controls found increased expression of IL-2 and IL-4 in smokers and in patients with oral lichen planus (a chronic inflammatory disease that affects the oral mucosa) [96,97]. Besides, increased salivary IL-4 also has been associated with periodontitis [97,98,99]. However, data about such salivary cytokines (TNF-β, IL-2, IL-4, and IL-13) in alcohol users were not found. A previous study with alcohol users assessed differences in salivary concentrations of TNF-α, IL-1α, and INF-γ compared to controls, showing significantly increased expression of all cytokines in alcohol users [100]. To the best of our knowledge, data from the current study provides new evidence identifying potential salivary biomarkers associated with alcohol use. The inflammatory profile revealed in the AG is different from other studies reporting that increases in serum/plasma TNF-α, IL-1, IL-6, IL-12, IL1-β, and IL-8 are associated with chronic alcohol consumption or alcohol-related liver diseases [16,101,102]. This difference might be due to genetics, age, cultural, and behavioral aspects (e.g., diet and lifestyle) [103].

Vitamin D deficiency in plasma was found in the CG, which has been described to be inversely associated with physiological dysregulation associated with chronic exposure to stress and adverse health outcomes [104]. Deficiency of vitamin D has also been found to independently predict cognitive impairment [105]. Although previous studies have related alcohol consumption with lower levels of vitamin D in plasma, we found higher levels of this vitamin in AG [106,107,108]. Nevertheless, vitamin D deficiency has been linked with other factors like inadequate sun exposure, limited oral intake, severe liver disease, renal insufficiency, and impaired intestinal absorption, among other risk factors [109]. A possible explanation is that some of the factors mentioned above may influence the level of vitamin D in our control group.

Although blood has been considered to be bacteria-free or ‘sterile’, a recent review describes that many bacteria can survive in a dormant form in blood in several non-communicable diseases such as diabetes mellitus and cardiovascular diseases [110]. A diversified microbiome has also been described in the blood of healthy donors [111]. The origin of these bacteria is suggested to be from the gut microbiota as well as the oral microbiota [110]. Leclercq et al. identified the alcohol-induced dysbiosis of gut microbiota and subsequent inflammatory responses as the likely causes of depressive disorders and alcohol craving [16]. Similarly, variation in the human oral microbiome caused by alcohol use has been related to systemic inflammatory and autoimmune disorders [112,113]. We found that the genus *Prevotella* was significantly more abundant in AG saliva. A similar finding was observed by Fan et al., who identified increased *Prevotella* in the oral microbiome of drinkers [113]. The observed differences in *Prevotella* among our groups may suggest a possible dysbiosis in the salivary microbiota of the alcohol users.

Interestingly, reduced executive functions were correlated to increased salivary concentrations of *Prevotella* in AG as well as in all samples. *Prevotella* has been documented as associated with periodontitis, cavities, oral cancer, and root canal procedures [114,115,116,117,118]. Other studies also have reported associations between oral diseases and reduced cognitive function [119,120,121]. To our knowledge, this is the first time that *Prevotella* is reported as inversely associated with executive function in the scenario of the oral microbiome and alcoholism. The few studies revealing a positive correlation between cognitive function and *Prevotella* assessed this bacteria in the gut and its antibody in serum [122,123]. Our study is possibly the first to report any correlation between oral *Prevotella* and cognitive function and in particular, to report a negative correlation.

## 5. Study Limitations

The small study sample size and the use of a case-control design limit the ability to draw firm conclusions. Therefore, a larger sample size, and a longitudinal design with more clearly defined and tightly controlled groups are needed. Possible confounders such as drug use, tobacco use, depression, anxiety, body mass index, and vitamin D deficiency may increase the inflammatory responses in the samples. Notably, the AG reported a higher frequency of tobacco use compared to the CG and might be confounding the study results. Another significant limitation of the study was the absence of a previous oral health status evaluation between AG and CG groups before sample collection. Periodontitis and dental caries, as well as other oral diseases that modulate the expression of pro-inflammatory cytokines, may drive cytokine expression between the groups [124]. The inclusion of a previous evaluation of the oral cavity in the current study could have increased the reliability of the results. Also, other factors in the methodology, such as the inclusion criteria for participants, sampling, storage conditions, and storage time before samples evaluation, may have some adverse effects in the assessment of cytokines concentrations [125].

## 6. Conclusions

The results of the present study support previous knowledge that alcohol use correlates with executive and immune system dysfunctions and dysbiosis of the salivary microbiota. Alcohol users showed upregulation of plasma MDC, confirming recent findings [91]. This study also identified potential alcohol-associated salivary inflammatory biomarkers (TNF-β, IL-2, IL-4, and IL-13), that may be important in its pathophysiology. Given the detrimental effects of alcohol in the individual’s mental and physiological health, these results warrant further investigation to understand the pathophysiology of alcohol use and mental comorbidities, and the use of salivary biomarkers in the identification of alcohol-related diseases.

## Figures and Tables

**Table 1:** Socio-demographic and clinical characteristics of study participants.

Parameter	Alcohol (n = 30)	Control (n = 20)	*p*-Value
Age in years (mean ± standard deviation)	41.6 ± 10.6	37.9 ± 14.5	0.124
	n (%)	n (%)	
Male	18 (60%)	10 (50%)	0.485
Unemployed	11 (37%)	10 (50%)	0.349
Retired	4 (13%)	4 (20%)	0.529
Lower education ^1^	13 (43%)	2 (10%)	0.815
Hispanic ethnicity	30 (100%)	20 (100%)	-
Tobacco smoker	19 (63%)	2 (10%)	0.0002 *
Use of drugs	11 (37%)	3 (15%)	0.095
Use of cannabis	9 (30%)	3 (15%)	0.054
Overweight/obese	22 (73%)	14 (70%)	0.797
Medical condition	18 (60%)	11 (55%)	0.726
Hypertension	7 (23%)	9 (45%)	0.797
Hypothyroidism	2 (7%)	4 (20%)	0.155
Asthma	4 (13%)	1 (5%)	0.336
Psychiatric disorder	14 (47%)	3 (15%)	0.021 *
Depression	9 (30%)	2 (10%)	0.035 *
Anxiety	11 (37%)	2 (10%)	0.035 *
Bipolar	1 (3%)	0 (0%)	0.409
Alcohol dependence	2 (7%)	0 (0%)	0.239
Sleep difficulties	24 (80%)	7 (35%)	0.001 *
Family history of alcoholism	22 (73%)	9 (45%)	0.043 *
Alcohol breath concentration (mean ± standard deviation)	0.006 ± 0.03	0.000 ± 0.00	0.243
AUDIT score (mean ± standard deviation)	20.77 ± 7.86	1.65 ± 1.31	0.000 *

^1^ High school or less; * Denotes statistically significant difference after performing a Mann–Whitney U test (continuous variables) or chi-square Test (categorical variables).

**Table 2:** Scores and severity in participant’s depression and social anxiety.

Questionnaire	Alcohol (n = 30)	Control (n = 20)	*p*-Value
PHQ-9 Score, Median (IQR)	8.5 (5.0–16.25)	4.0 (2.0–8.0)	0.007 *
Depression Symptoms Severity Level	n (%)	n (%)	
Minimal	5 (17%)	12 (60%)	
Mild	12 (40%)	5 (25%)	
Moderate	5 (17%)	1 (5%)	
Mod-severe	5 (17%)	1 (5%)	
Severe	3 (10%)	1 (5%)	
LSAS-SR Score, Median (IQR)	18.5 (8.0–36.3)	24.0 (14.3–35.5)	0.238
Social Phobia Severity Level	n (%)	n (%)	
Negligible	29 (97%)	18 (90%)	
Moderate	1 (3%)	0 (0%)	
Marked	0 (0%)	2 (10%)	

* Denotes statistically significant difference after performing a Mann–Whitney U test.

**Table 3:** Scores on selected measures of NIH toolbox cognition battery.

		Alcohol		Control
	n	Median (IQR)	n	Median (IQR)
Cognitive composite ^1^	28	75.5 (65.3–92.0)	19	100.0 (91.0–115.0) *
Attention and executive function ^1^				
Flanker inhibitory	28	82.5 (77.0–98.8)	19	103.0 (86.0–115.0) *
Dimensional change card sort	29	95.0 (84.0–103.0)	19	107.0 (103.0–114.0) *

^1^ Fully adjusted standard score. * Denotes statistically significant difference after performing a Mann–Whitney U test, *p* ≤ 0.001.

**Table 4:** Cytokine levels in the saliva and plasma samples.

**Salivary Cytokines**	**Alcohol** **pg/mL** **Median (IQR)**	**Control ^1^** **pg/mL** **Median (IQR)**
TNF-alpha	10.4 (2.7–26.6)	4.9 (1.7–27.3)
IL-12p70	2.9 (2.3–7.1)	8.7 (3.3–12.7) *
MDC	141.0 (46.1–397.3)	185.0 (91.7–324.0)
IL-10	3.3 (3.3–3.3)	1.7 (1.7–5.9)
INF-γ	2.7 (2.7–6.9)	4.9 (1.7–6.9)
TNF-β	4.8 (4.8–4.8)	1.9 (1.9–3.5) **
IL-1β	52.4 (5.8–93.3)	21.7 (5.7–209.0)
IL-5	1.2 (.9–1.5)	2.6 (1.7–3.3) **
IL-2	1.9 (1.9–1.9)	1.1 (1.1–1.1) **
IL-6	14.2 (5.1–32.9)	7.4 (3.2–21.4)
IL-4	4.2 (4.2–5.0)	1.1 (1.1–1.1) **
IL-13	3.1 (3.1–3.1)	1.4 (1.4–1.9) **
IL-17A	1.9 (1.7–4.6)	3.5 (1.4–4.6)
IL-7	10.1 (2.0–22.5)	8.2 (5.4–12.8)
GM-CSF	10.6 (4.0–20.5)	15.8 (4.6–34.3)
**Plasma Cytokines**	**Alcohol**	**Control**
TNF-alpha	20.4 (14.6–28.4)	15.92 (8.3–26.4)
IL-12p70	54.7 (13.3–164.8)	23.1 (4.4–52.1)
MDC	1223.0 (840.8–1789.0)	852.0 (702.3–1031.0) *
IL-10	9.0 (5.7–16.9)	10.4 (4.5–16.3)
INF-γ	79.8 (27.9–141.8)	43.4 (20.2–71.6)
TNF-β	9.9 (1.9–38.1)	16.0 (2.2–118.5)
IL-1β	6.1 (2.4–24.1)	7.0 (2.4–17.3)
IL-5	3.3 (2.2–7.9)	3.4 (1.6–10.6)
IL-2	4.0 (1.7–24.6)	4.8 (2.3–12.9)
IL-6	8.4 (2.5–20.2)	8.9 (2.2–42.7)
IL-4	2.3 (2.3–34.7)	2.3 (1.1–8.3)
IL-1RA	51.8 (38.1–88.7)	50.7 (42.0–87.6)
IL-13	7.2 (2.3–24.9)	14.2 (4.5–41.7)
IL-17A	41.4 (20.4–112.0)	23.9 (11.3–44.3)
IL-7	3.6 (1.9–7.0)	3.0 (0.6–4.9)
GM-CSF	35.7 (13.6–65.9)	35.0 (7.0–68.2)

^1^ The analysis of salivary cytokines was performed on 19 samples. Significant differences observed after conducting a Mann–Whitney U Test, * *p* ≤ 0.01, ** *p* ≤ 0.001.

**Table 5:** Concentrations of cortisol, insulin, leptin, and vitamin-D in the saliva and plasma of the alcohol and control groups.

**Saliva**	**Alcohol** **Median (IQR)**	**Control ^1^** **Median (IQR)**
Insulin (pg/mL)	246.5 (124.3–643.5)	143.0 (60.9–371.0)
Leptin (pg/mL)	28.7 (15.0–43.1)	25.2 (18.2–44.3)
Cortisol (ng/mL)	15.9 (9.7–42.8)	22.8 (13.8–37.8)
Vitamin D (ng/mL)	11.9 (6.9–23.8)	60.0 (43.2–78.9) **
**Plasma**	**Alcohol ^2^** **Median (IQR)**	**Control ^3^** **Median (IQR)**
Insulin (pg/mL)	265.0 (105.7–487.0)	213.5 (95.9–431.0)
Leptin (pg/mL)	9237.0 (3901.0–15601.5)	3807.5 (1681.3–17824.0)
Cortisol (ng/mL)	1.64 (0.44–15.9)	3.5 (0.62–10.4)
Vitamin D (ng/mL)	45.78 (30.2–81.4)	18.4 (11.9–63.4) *

^1^ The analysis of saliva concentrations for insulin and leptin in CG were performed with 19 samples. ^2^ The analysis of plasma concentrations for insulin and leptin in the AG were performed with 29 samples. ^3^ The analysis of plasma concentrations for cortisol in the CG was performed with 18 samples. Differences between groups were determined using a Mann–Whitney U test, * *p* ≤ 0.01, ** *p* ≤ 0.001.

## Supplementary Materials

The following are available online at https://www.mdpi.com/1660-4601/17/3/689/s1. Table S1: Nutrients consumed based on reported dietary intake in the last month.
