# Supplementary material for: Inflammatory Biomarkers, Microbiome, Depression, and Executive Dysfunction in Alcohol Users
Source: Int J Environ Res Public Health. 2020 Jan 21;17(3):689. doi: 10.3390/ijerph17030689 (PMC7037324; doi:10.3390/ijerph17030689)
Supplement: Supplementary file 1 [file ijerph-17-00689-s001.pdf]

Supplementary Table 1. Nutrients consumed based on reported dietary intake in the last month.

Nutrient	All Samples (n = 50) Median (IQR)	Alcohol (n = 30) Median (IQR)	Control (n = 20) Median (IQR)	*p*-value
Acesulfame Potassium (mg)	0.00 (0.0-0.0)	0.00 (0.0-0.0)	0.00 (0.0-0.0)	0.736
Added Sugars (gm)	40.21 (19.1-85.7)	43.79 (18.7-99.7)	40.21 (19.4-78.9)	0.766
Alanine (gm)	3.30 (1.6-4.9)	40.04 (16.7-90.2)	36.63 (17.3-70.6)	0.707
Alcohol (gm)	7.13 (0.4-32.2)	16.75 (7.9-56.9)	0.04 (0.02-1.84)	0.000*
Alpha-Carotene (mcg)	223.69 (118.7-649.7)	205.12 (70.2-780.1)	265.58 (146.2-511.8)	0.874
Alpha-toc eq (mg)	7.14 (4.3-10.2)	7.56 (3.5-12.9)	6.91 (5.1-8.4)	0.797
Apha-Tocopherol (mg)	5.59 (3.5-8.0)	5.82 (2.8-9.6)	5.59 (3.9-7.1)	0.843
Arginine (gm)	3.54 (1.7-5.1)	3.57 (1.4-5.4)	3.54 (2.4-5.0)	0.579
Ash (gm)	15.55 (9.4-23.7)	14.97 (9.4-26.9)	17.69 (9.1-23.5)	0.968
Aspartame (mg)	0.00 (0.0-10.9)	0.00 (0.00-5.5)	1.23 (0.00-15.3)	0.177
Aspartic acid (gm)	6.18 (2.9-9.5)	6.36 (2.6-9.5)	5.97 (3.9-9.7)	0.707
Beta-Carotene (mcg)	1363.68 (852.9-3374.8)	996.96 (708.2-3563.4)	1644.50 (919.2-3154.9)	0.452
Beta-Cryptoxantin (mcg)	42.66 (18.6-93.8)	42.66 (16.8-94.4)	41.45 (21.2-96.3)	0.890
Betaine (mg)	124.52 (83.4-209.1)	150.9 (92.8-238.0)	105.23 (74.2-176.6)	0.227
Beta-Tocopherol (mg)	0.33 (0.2-0.5)	0.30 (0.18-0.61)	0.33 (0.23-0.39)	0.782
Biochanin A (mg)	0.004 (0.001-0.007)	0.004 (0.001-0.007)	0.004 (0.001-0.007)	0.812
Caffeine (mg)	62.38 (17.8-230.7)	80.03 (17.8-215.1)	56.57 (12.6-253.1)	0.859
Calcium (mg)	702.56 (433.7-1143.6)	588.21 (433.87-1461.6)	866.36 (429.2-1097.9)	0.452
Energy (Kcal)	1747.47 (1134.9-2990.7)	1747.47 (1155.4-3271.9)	1669.15 (1025.9-2350.6)	0.313
Total Carbohydtate (gm)	195.63 (127.6-335.8)	200.92 (128.2-408.3)	183.09 (118.1-313.3)	0.406
Chloesterol (mg)	240.54 (97.9-342.7)	212.43 (83.4-358.4)	275.47 (168.9-343.9)	0.243
Choline (mg)	313.66 (177.3-545.6)	312.80 (165.2-600.7)	318.53 (181.2-454.5)	0.890
CLA cis-9, trans-11 (gm)	0.08 (0.05-0.14)	0.08 (0.05-0.15)	0.08 (0.06-0.12)	0.722
CLA trans-10, cis-12 (gm)	0.02 (0.01-0.03)	0.02 (0.01-0.03)	0.02 (0.01-0.03)	0.890
Copper (gm)	1.16 (0.6-1.5)	1.16 (0.6-1.6)	1.14 (0.6-1.3)	0.552
Coumestrol (mg)	0.06 (0.02-0.12)	0.07 (0.02-0.12)	0.06 (0.02-0.13)	0.507
Cystine (gm)	0.87 (0.4-1.2)	0.87 (0.4-1.5)	0.87 (0.6-1.2)	0.607
Daidzein (mg)	0.13 (0.5-0.3)	0.13 (0.04-0.25)	0.13 (0.8-0.29)	0.859
Delta-Tocopherol (mg)	2.19 (1.3-3.5)	2.10 (1.2-3.9)	2.19 (1.4-3.2)	0.766
Erythitol (g)	0.0002 (0.0001-0.0005)	0.0001 (0.0000-0.0005)	0.0003 (0.0001-0.0007)	0.178
Total Fat (gm)	58.34 (34.0-89.7)	58.13 (28.9-103.8)	58.34 (45.6-77.1)	0.874
Dietary Fiber (gm)	12.61 (7.4-19.3)	12.20 (5.9-12.6)	13.45 (7.5-18.5)	0.937
Water Sol Dietary Fiber (gm)	4.44 (2.52-6.57)	4.59 (2.4-8.2)	4.04 (2.7-6.4)	0.722
Insoluble Dietary Fiber (gm)	7.42 (4.6-12.9)	7.31 (3.9-13.3)	8.55 (4.7-12.6)	0.812
Folate-dietary equivalents (mcg)	391.01 (264.5-577.7)	391.01 (250.8-582.6)	391.51 (271.9-563.2)	0.984
Folate-natural (food folate) (mcg)	182.02 (100.7-263.0)	180.23 (111.6-264.8)	184.57 (100.4-226.6)	0.678
Folate-synthetic (folic acid) (mcg)	120.30 (87.7-193.5)	121.09 (77.4-203.2)	120.30 (101.9-187.9)	0.649
Formononetin (mg)	0.0003 (0.0001-0.0007)	0.0003 (0.0000-0.0007)	0.0004 (0.0001-0.0007)	0.789
Fructose (gm)	17.90 (9.14-36.65)	18.68 (8.9-56.2)	16.68 (10.3-27.5)	0.579
Galactose (gm)	0.31 (0.1-0.6)	0.31 (0.08-0.63)	0.30 (0.11-0.54)	0.890
Gamma-Tocopherol (mg)	10.69 (5.9-16.7)	11.21 (5.7-21.5)	10.69 (5.9-14.5)	0.440
Genistein (mg)	0.15 (0.1-0.3)	0.14 (0.05-0.21)	0.15 (0.06-0.33)	0.440

Glucose (gm)	18.97 (9.2-35.2)	19.03 (9.1-56.9)	17.34 (10.5-29.9)	0.722
Glutamic acid (gm)	13.79 (7.1-21.3)	13.12 (6.7-21.3)	14.11 (8.9-20.4)	0.678
Glycine (gm)	2.80 (1.31-3.93)	3.05 (1.1-4.2)	2.70 (1.9-3.9)	0.736
Glycitein (gm)	0.009 (0.005-0.025)	0.007 (0.005-0.018)	0.013 (0.007-0.033)	0.137
Gram Amount	3170.60 (1575.9-4648.5)	3273.92 (2056.4-5573.9)	2972.84 (1320.8-3916.9)	0.259
Histidine (gm)	1.92 (0.8-2.9)	1.93 (0.8-3.0)	1.91 (1.3-2.8)	0.526
Inositol (g)	0.10 (0.03-0.20)	0.12 (0.02-0.28)	0.07 (0.03-0.16)	0.406
Iron (mg)	10.18 (6.8-16.7)	9.64 (5.8-17.6)	11.38 (7.0-14.9)	0.953
Isoleucine (gm)	3.19 (1.4-4.9)	3.23 (2.0-4.7)	3.16 (2.0-4.7)	0.579
Isomalt (g)	0.00 (0.0-0.0)	0.00 (0.0-0.0)	0.00 (0.0-0.0)	1.000
Energy (kj)	7311.41 (4748.5-12513.3)	7311.41 (48.34.3-13689.9)	6983.71 (4292.8-9834.8)	0.313
Lactilol (g)	0.00 (0.0-0.0)	0.00 (0.0-0.0)	0.00 (0.0-0.0)	1.000
Lactose (gm)	11.58 (6.5-22.8)	10.91 (7.6-25.4)	12.70 (5.2-24.7)	0.968
Leucine (gm)	3.39 (2.5-8.4)	5.38 (2.3-8.5)	5.47 (3.4-8.2)	0.464
Lutein + Zeaxanthin (mcg)	814.50 (465.4-1501.5)	859.29 (442.3-1501.5)	803.04 (491.7-1559.3)	0.968
Lycopene (mcg)	4403.72 (2027.9-6196.2)	4544.85 (1992.9-6345.5)	4094.10 (2109.7-5736.1)	0.722
Lysine (gm)	4.59 (2.1-7.3)	4.56 (1.9-7.7)	4.59 (2.9-7.1)	0.539
Magnesium (mg)	255.08 (150.4-437.5)	240.66 (159.6-465.0)	294.12 (139.9-386.7)	0.751
Maltitol (g)	0.00 (0.0-0.0)	0.00 (0.0-0.0)	0.00 (0.0-0.0)	0.110
Maltose (gm)	2.41 (1.1-3.8)	2.36 (0.9-4.3)	2.41 (1.4-3.6)	0.797
Manganese (mg)	2.31 (1.4-3.5)	2.32 (1.2-4.2)	2.34 (1.5-3.5)	0.937
Mannitol (g)	0.15 (0.09-0.23)	0.14 (0.8-0.2)	0.15 (0.1-0.2)	1.000
Methylhistidine (mg)	11.78 (5.2-21.7)	9.91 (4.1-22.3)	12.35 (6.9-17.9)	0.394
Methionine (gm)	1.58 (0.7-2.5)	1.52 (0.6-2.6)	1.58 (1.0-2.4)	0.488
MFA 14:1 (gm)	0.07 (0.04-0.13)	0.08 (0.03-0.14)	0.07 (0.05-0.11)	0.843
MFA 16:1 (gm)	0.90 (0.5-1.4)	0.90 (0.4-1.4)	0.94 (0.6-1.4)	0.678
MFA 18:1, oleic acid (gm)	19.53 (11.6-29.6)	21.23 (9.9-34.8)	19.09 (15.5-26.6)	0.843
MFA 20:1 (gm)	0.21 (0.12-0.31)	0.21 (0.10-0.34)	0.20 (0.15-0.26)	0.921
MFA 22:1 (gm)	0.007 (0.004-0.028)	0.007 (0.003-0.016)	0.007 (0.004-0.056)	0.303
Total MFA (gm)	21.02 (12.4-32.1)	22.45 (10.7-37.7)	20.45 (16.5-28.9)	0.905
Natural Alpha-Tocopherol (mg)	5.59 (3.5-7.7)	5.80 (2.8-8.7)	5.59 (3.7-7.0)	0.736
Niacin (mg)	18.61 (13.1-31.8)	20.14 (13.1-35.0)	10.47 (12.8-23.2)	0.384
Niacin Equivalents (mg)	34.55 (20.1-51.8)	33.19 (19.4-58.6)	34.84 (21.4-44.0)	0.812
Nitrogen (mg)	11.43 (5.4-18.1)	11.50 (5.3-18.5)	11.40 (7.3-17.4)	0.797
Omega-3 Fatty Acids (mg)	1.56 (0.8-2.1)	1.62 (0.8-2.1)	1.32 (0.8-1.9)	0.488
Oxalic Acid (mg)	111.14 (67.3-169.6)	122.35 (64.3-184.5)	94.86 (80.1-162.3)	0.607
Panthoteic Acid (mg)	4.26 (2.49-7.43)	4.15 (2.5-9.0)	4.31 (2.3-7.2)	0.953
Pectins (gm)	1.39 (0.7-2.2)	1.50 (0.7-2.2)	1.26 (0.7-2.2)	0.937
PFA 18:2, linoleic acid (gm)	11.15 (6.4-15.5)	11.62 (5.9-19.5)	11.13 (7.1-13.2)	0.635
PFA 18:3, linoleic acid (gm)	1.47 (0.7-1.9)	1.54 (0.7-2.0)	1.27 (0.7-1.8)	0.322
PFA 18:3N3 (gm)	1.41 (0.7-1.9)	1.49 (0.6-2.0)	1.23 (0.6-1.7)	0.342
PFA 18:4 (gm)	0.002 (0.0004-0.0059)	0.0014 (0.0003-0.0038)	0.0032 (0.0007-0.0147)	0.109
PFA 20:4 (gm)	0.10 (0.05-0.17)	0.09 (0.04-0.18)	0.11 (0.08-0.16)	0.417
PFA 20:5, EPA (gm)	0.02 (0.01-0.05)	0.019 (0.006-0.046)	0.027 (0.009-0.066)	0.166
PFA 22:5 (gm)	0.01 (0.007-0.029)	0.014 (0.005-0.029)	0.015 (0.010-0.031)	0.243

PFA 22:6, DHA (gm)	0.05 (0.02-0.11)	0.05 (0.02-0.10)	0.06 (0.03-0.15)	0.285
Total PFA (gm)	12.87 (7.3-18.3)	13.44 (7.0-22.2)	12.59 (7.9-15.4)	0.593
Phenylalanine (gm)	3.07 (1.4-4.7)	2.98 (1.3-4.7)	3.14 (1.9-4.7)	0.526
Phosphorous (mg)	1135.58 (594.1-1679.2)	1031.82 (576.5-1738.7)	1225.89 (624.8-1650.9)	0.751
Phyric Acid (mg)	450.98 (281.3-705.6)	422.27 (223.7-737.8)	467.28 (316.9-640.8)	0.812
Pinitol (g)	0.006 (0.001-0.027)	0.005 (0.001-0.026)	0.008 (0.002-0.036)	0.722
Potassium (mg)	2269.24 (1245.3-4008.9)	2166.82 (1245.3-4294.4)	2331.52 (1185.2-3379.8)	0.812
Proline (gm)	4.65 (2.5-7.0)	4.36 (2.4-7.4)	4.85 (2.8-6.5)	0.782
Animal protein (gm)	47.82 (20.9-75.6)	43.21 (16.8-83.2)	47.82 (29.2-70.4)	0.513
Protein (gm)	70.66 (33.1-110.7)	71.4 (32.6-114.3)	70.39 (44.5-106.4)	0.751
Vegetable protein (gm)	18.9 (14.0-28.6)	20.23 (10.3-24.1)	18.69 (15.1-30.9)	0.607
Retinol (mcg)	414.32 (253.0-662.0)	371.92 (230.9-831.6)	431.8 (315.7-614.4)	0.663
Riboflavin (mg)	1.66 (1.0-2.7)	1.58 (1.1-3.7)	1.78 (0.9-2.5)	0.905
Saccharin (mg)	0.00 (0.0-0.0)	0.00 (0.0-0.0)	0.00 (0.0-0.0)	1.000
Synthetic Alpha-Tocopherol (mg)	0.04 (0.0-0.2)	0.03 (0.00-0.10)	0.05 (0.00-0.29)	0.496
Selenium (mcg)	96.18 (57.2-156.9)	97.37 (51.2-159.2)	96.18 (71.7-141.1)	0.692
Serine (gm)	3.10 (1.5-4.8)	3.00 (1.4-4.8)	3.29 (1.9-4.9)	0.513
SFA 10:0 (gm)	0.33 (0.19-0.54)	0.32 (0.2-0.6)	0.36 (0.2-0.5)	0.751
SFA 12:0 (gm)	0.54 (0.2-1.1)	0.55 (0.3-1.2)	0.52 (0.3-1.1)	0.953
SFA 14:0 (gm)	1.42 (0.9-2.9)	1.35 (0.9-3.2)	1.55 (1.1-2.0)	0.539
SFA 16:0, palmitic acid (gm)	9.87 (5.9-16.4)	9.49 (5.1-19.1)	10.55 (7.6-13.5)	0.890
SFA 17:0 (gm)	0.09 (0.06-0.15)	0.09 (0.05-0.18)	0.09 (0.07-0.12)	0.552
SFA18:0, stearic acid (gm)	4.49 (2.7-7.4)	4.08 (2.4-9.3)	4.88 (3.2-6.0)	0.968
SFA 20:0 (gm)	0.11 (0.06-0.18)	0.14 (0.06-0.23)	0.11 (0.9-0.14)	0.294
SFA 22:0 (gm)	0.07 (0.03-0.11)	0.07 (0.03-0.13)	0.07 (0.05-0.09)	0.921
SFA 4:0 (gm)	0.35 (0.24-0.63)	0.34 (0.20-0.73)	0.40 (0.26-0.57)	0.692
SFA 6:0 (gm)	0.21 (0.11-0.31)	0.20 (0.1-0.4)	0.22 (0.1-0.3)	0.751
SFA 8:0 (gm)	0.19 (0.9-0.30)	0.19 (0.1-0.3)	0.18 (0.1-0.3)	0.782
Total SFA (gm)	18.02 (11.1-34.4)	16.67 (9.8-36.7)	20.10 (13.9-23.9)	0.859
Sodium (mg)	2632.30 (1669.5-4087.5)	2691.71 (1406.7-4316.2)	2632.30 (1756.5-4069.2)	0.782
Solid Fat (g)	25.15 (14.9-44.4)	21.72 (12.3-53.8)	27.65 (17.4-37.3)	0.663
Sorbitol (g)	0.18 (0.1-0.4)	0.21 (0.1-0.6)	0.18 (0.0-0.4)	0.464
Starch (gm)	72.83 (42.1-129.1)	70.99 (41.5-131.1)	76.01 (43.1-106.2)	0.890
Sucrose polyester (gm)	0.01 (0.00-0.04)	0.01 (0.00-0.4)	0.01 (0.00-0.06)	0.646
Sucralose (mg)	0.00 (0.0-0.0)	0.00 (0.0-0.0)	0.00 (0.0-0.0)	1.000
Sucrose (gm)	26.21 (14.8-54.7)	27.27 (13.7-64.4)	26.21 (15.9-42.9)	0.722
Tagatose (mg)	1.02 (0.5-2.5)	0.85 (0.5-4.4)	1.26 (0.6-2.1)	0.828
TRANS 16:1 (gm)	0.03 (0.02-0.05)	0.03 (0.01-0.05)	0.03 (0.02-0.05)	0.513
TRANS 18:1 (gm)	1.81 (0.9-2.9)	1.49 (0.9-3.5)	2.02 (1.0-2.3)	0.968
TRANS 18:2 (gm)	0.28 (0.16-0.47)	0.23 (0.15-0.7)	0.32 (0.18-0.4)	1.000
Total Grain (g)	4.69 (2.9-8.1)	4.69 (2.7-9.2)	4.71 (3.3-7.4)	0.905
Thiamin (mg)	1.32 (0.8-2.0)	1.20 (0.7-2.1)	1.47 (0.9-2.0)	0.984
Threonine (gm)	2.73 (1.2-4.2)	2.73 (1.2-4.3)	2.71 (1.7-4.1)	0.579
Total Trans-Fatty Acids (gm)	2.19 (1.1-3.6)	1.79 (1.1-4.3)	2.50 (1.2-2.8)	0.968
Total Conjugated Linoleic Acid (gm)	0.10 (0.06-0.18)	0.10 (0.05-0.19)	0.09 (0.07-0.15)	0.751
Total Folat (mcg)	314.46 (205.9-443.8)	304.10 (212.6-443.8)	320.23 (193.6-463.2)	0.984

Total Sugar (gm)	74.03 (45.6-186.2)	90.27 (44.7-189.6)	69.63 (48.3-171.6)	0.692
Tryptophan (gm)	0.79 (0.4-1.1)	0.78 (0.3-1.1)	0.79 (0.5-1.2)	0.621
Tyrosine (gm)	2.40 (1.1-3.6)	2.38 (1.0-3.6)	2.40 (1.4-3.6)	0.513
Valine (gm)	3.55 (1.6-5.5)	3.51 (1.5-5.5)	3.60 (2.2-5.4)	0.488
Total Vit A (international units)	4505.01 (2494.2-8606.2)	3686.98 (2174.6-9387.5)	5100.58 (3731.1-8026.6)	0.406
Total Vit A (retinol activity equiv)	574.70 (359.3-960.1)	517.44 (334.6-1076.9)	614.23 (426.2-938.0)	0.566
Total Vit A (retinol equiv)	766.8 (443.5-1285.0)	649.73 (383.5-1374.8)	783.33 (595.2-1292.2)	0.440
Vitamin B12 (mcg)	5.37 (2.5-7.2)	5.12 (2.3-7.9)	5.42 (3.1-6.8)	0.751
Vitamin B6 (mg)	1.51 (1.0-2.3)	1.45 (1.0-2.6)	1.63 (0.9-2.1)	0.649
Vitamin C (mg)	54.65 (27.7-100.8)	64.57 (20.2-124.6)	47.58 (29.0-72.1)	0.692
Vitamin D (mcg)	4.68 (2.6-10.3)	3.89 (2.4-7.8)	5.93 (3.3-10.5)	0.205
Vitamin D2 (mcg)	0.001 (0.0008-0.003)	0.001 (0.0008-0.0024)	0.001 (0.0008-0.0033)	0.607
Vitamin D3 (mcg)	4.68 (2.6-10.3)	3.89 (2.4-7.8)	5.92 (3.3-10.5)	0.205
Vitamin E (international units)	8.58 (5.2-12.1)	8.69 (4.3-15.0)	8.58 (5.9-10.5)	0.905
Vitamin K (mcg)	63.43 (41.2-115.2)	66.45 (38.8-152.8)	61.56 (41.9-79.5)	0.373
Water (gm)	2714.72 (1378.5-4175.4)	2838.78 (1872.4-4845.8)	2657.88 (1109.6-3529.3)	0.294
Whole Grain (g)	0.81 (0.3-1.9)	0.62 (0.2-1.5)	0.93 (0.4-1.9)	0.243
Xylitol (g)	0.007 (0.004-0.014)	0.009 (0.004-0.035)	0.006 (0.004-0.010)	0.267
Zinc (mg)	9.05 (4.9-13.4)	8.43 (3.9-15.7)	9.63 (5.7-11.4)	0.707

* Denotes statistically significant difference after performing a Man-Whitney U Test.
